# Vector competence of *Aedes albopictus* for Tonate virus highlights transmission risks in temperate and tropical regions

**DOI:** 10.1080/22221751.2025.2547733

**Published:** 2025-08-14

**Authors:** Isabelle Moltini-Conclois, Wipaporn Khanom, Elliott F. Miot, Ai-rada Pintong, Hermann Landry Munshili Njifon, Carole Ginebre, Valérie Choumet, Bruno Coutard, Cédric Mariac, Pierre Roques, Agathe M. G. Colmant, Géraldine Piorkowski, Julien Pompon, Dorothée Missé

**Affiliations:** a MIVEGEC, Univ. Montpellier, CNRS, IRD, Montpellier, France; b Annex of Garoua, Centre Pasteur of Cameroon, Garoua, Cameroon; c Institut Pasteur, Environnement et Risques Infectieux, Université Paris Cité, Paris, France; d Unité Des Virus Emergents (UVE: Aix-Marseille Univ, Universita Di Corsica, IRD 190, Inserm 1207, IRBA), Marseille, France; e DIADE, Univ Montpellier, CIRAD, IRD, Montpellier, France; f Virology Unit, Institut Pasteur de Guinée, Conakry, Guinea; g Institute of Molecular Biosciences, Mahidol University, Thailand; h Department of Medical Technology, LMI PRESTO, Faculty of Associated Medical Sciences, Chiang Mai University, Chiangmai, Thailand

**Keywords:** Arbovirus, Tonate virus, Chikungunya virus, *Aedes albopictus*, vector competence

## Abstract

Recent evidence of vertical transmission of Tonate virus (TONV) during early pregnancy and its association with fetal neurological anomalies highlights its potential public health threat. TONV is an understudied alphavirus endemic to French Guiana. The growing presence of *Aedes (Ae.) albopictus* in Europe raises concerns about its ability to transmit emerging arboviruses, including TONV. We assessed the vector competence of *Ae. albopictus* populations from mainland France and La Réunion Island via oral infections using different TONV doses. Both populations supported efficient viral replication, with infectious viral particles appearing in saliva by day 5 post-infection. Infection rate (IR), stepwise dissemination rate (sDR), and transmission efficiency (TE) increased with rising viral concentrations. At a viral concentration of 10^6^ PFU/mL IR reached 80%, and TE at day 5 post-infection was 27% for the mainland strain and 37% for the La Réunion strain. Notably, TE declined over time in the mainland strain, while increasing progressively in the La Réunion population. Comparative infections with Chikungunya virus revealed that TONV was transmitted at similar or greater rates, confirming *Ae. albopictus* as a competent vector. Sequencing of mosquito organs revealed intra-host TONV genetic variability. A recurrent polymorphism at position 11,357 in the 3′UTR was detected in body tissues but not in heads of La Réunion mosquitoes, suggesting tissue-specific selection or bottlenecks. Our results demonstrate that *Ae. albopictus* from both tropical and temperate areas can efficiently transmit TONV and emphasize the importance of genomic surveillance to anticipate risk of its emergence in areas where this vector is established.

## Introduction

Tonate virus (TONV), a member of the *Alphavirus* genus within the *Togaviridae* family, is classified under subtype IIIb of the Venezuelan equine encephalitis complex [1]. First identified in 1973 in a bird species (*Psarocolius decumanus*) in the village of Tonate, French Guiana, TONV has since been associated with human cases [2]. Despite its long-standing presence, TONV remains poorly characterized, with only a limited number of seroprevalence studies providing insights into its epidemiological patterns [3,4]. These studies have revealed significant geographic variation in seroprevalence rates, ranging from 0% to 55%. Notably, higher anti-TONV IgG rates (42–53%) have been observed in communities near border rivers in French Guiana compared to coastal areas (0–10%) [3]. To date, no imported or autochthonous human cases of TONV have been reported outside French Guiana. This absence likely reflects underdiagnosis, as TONV causes dengue-like symptoms that are easily mistaken for infections with co-circulating arboviruses such as Dengue virus (DENV), Zika virus (ZIKV), or Chikungunya virus (CHIKV) [4]. In the absence of specific diagnostic tools, infections may go undetected, underscoring the need for improved awareness and surveillance. Although the clinical spectrum of TONV infection remains poorly described, the virus has been associated with febrile syndromes and, in rare cases, encephalitis, suggesting a neurotropic potential [5]. Alarmingly, vertical transmission of TONV during early pregnancy has been linked to fetal neurological abnormalities, further highlighting the virus’s relevance to public health [6]. TONV is a zoonotic virus with a broad host range, including various bird species such as Passeriformes like house sparrows (*Passer domesticus*) or Crested Oropendola (*Psarocolius decumanus)*, swallows (*Petrochelidon pyrrhonota*) [7]. A Recent study have expanded its known wild mammals host spectrum to bats, including species such as *Trachops cirrhosus* and *Desmodus rotundus*, underscoring the ecological complexity of TONV [8].

Tonate virus is primarily maintained in a sylvatic cycle involving *Culex* (Melanoconion) and *Anopheles* mosquitoes, as well as phlebotomine sand flies (*Lutzomyia* spp.), from which the virus has been isolated in French Guiana [9–11]. Given the limited number of studies on TONV vectors, the involvement of other arthropods remains to be elucidated, including those known to be responsible for the widespread transmission of arboviruses, such as *Aedes (Ae.) albopictus* and *Ae. aegypti*.

The increasing prevalence of *Ae. albopictus*, an invasive mosquito species, presents a growing concern for the spread of arboviruses in Europe and beyond [12,13]. Originally native to Asia, *Ae. albopictus* is now established in 71 departments in France and is well-known for its ability to transmit arboviruses such as dengue, chikungunya, and Zika. Its remarkable adaptability to diverse environments, coupled with climate-driven expansion, extends the risk of arboviral outbreaks in previously unaffected regions [14]. However, its role as a potential vector for TONV has not been previously investigated, leaving a critical gap in mapping the epidemiological risk associated with this emerging alphavirus in France.

Despite its initial isolation over five decades ago and the regular detection of TONV in febrile patients in French Guiana, the virus remains poorly characterized from an entomological and epidemiological standpoint. Its confirmed presence in *Culex*, *Anopheles*, and *Lutzomyia* vectors suggests a complex sylvatic transmission cycle, but the potential for urban and peri-urban spillover has not been adequately explored. Given the expanding geographic range and anthropophilic behaviour of *Ae. albopictus*, a mosquito now established across much of Europe and known for its vector competence for several alphaviruses [14], it is crucial to determine whether this species could act as a good vector for TONV. To our knowledge, no data are available on the ability of *Ae. albopictus* to transmit TONV. Filling this gap is essential to assess the epidemic potential of this neglected arbovirus in regions where *Ae. albopictus* is abundant and human populations may be immunologically naive. To assess whether *Ae. albopictus* is a competent vector for TONV, we tested its ability to support viral progression to the salivary glands, a prerequisite for transmission during a subsequent bite [15].

## Materials and methods

### Cell culture, virus and reagents

VeroCCL81, African green monkey kidney cells (Sigma, France) were used. Cells were cultured in Dulbecco's Modified Eagle Medium (DMEM; Invitrogen, France) with 10% heat-inactivated FBS (Eurobio Scientific, France) and 1% penicillin–streptomycin (Gibco). The Tonate virus CaAn 410d strain (CRORA collection, Institut Pasteur, Paris) was isolated from a *Psarocolius decumanus* (Crested oropendola) in French Guiana, following passage in newborn mice in 1973. The virus was then retrieved and passaged nine times on VERO cells. It was sequenced using Oxford Nanopore MinION. Library prep used the Direct cDNA sequencing kit (SQK-LSK114) on 670 ng cDNA. Sequencing yielded 266 Mb (84 Kreads); consensus (99.95% identity) was deposited in GenBank (PV600894). Chikungunya virus (CHIKV, OPY1 strain, La Réunion Island 2006) was isolated from a viremic patient and provided by the European Virus Archive Global.

### Mosquito rearing

*Ae. albopictus* La Réunion (gift from Dr. Gouagna, IRD, Montpellier) F6 generation colony and *Ae. albopictus* France F5 generation colony (IRD, L1 virus-free Laboratory, Vectopole Montpellier,) were used. A total of 400 larvae per tray were reared in filtered water with Novo Prawn® fish food. Adults were maintained at 27°C, 80% humidity, 12:12 L:D cycle, with 10% sucrose.

### Mosquito infection

Experiments were conducted at BSL-3 Vectopole, under French/European regulations (IRD E34172221). Females aged 4–7 days were starved before receiving a 3 mL blood meal (1/3 virus, 2/3 washed rabbit blood from IRD) (DAP n°2212-50999). The mix contained DMEM, 10% FBS, 1% sodium bicarbonate, and 5 mM ATP. TONV titers were 10^5^, 10^6^, or 10^7^ PFU/mL. Feeding was done via Hemotek® with porcine membrane. Fully engorged females were kept at 27˚C, 80% humidity, 12:12 L:D with 10% sucrose. For each of the four time points post-infection (days 5, 10, 15, and 20), three groups of 60 mosquitoes were blood-fed with the viral doses described above to obtain 30 fully engorged individuals per condition for analysis.

### Vector competence indices

Saliva was collected at 5-, 10-, 15-, and 20- days post-infection (dpi) as previously described [15]. Legs/wings removed; proboscis was inserted into a tip with 5μL FBS. After 30 min, saliva was expelled into 45μL DMEM and frozen. Bodies and heads were dissected from each individual female mosquito at the different days post-infection(dpi) and stored at −80°C in DMEM with FBS and antibiotics. Organs and saliva from ∼30 mosquitoes per condition were titrated to calculate infection rate (IR), stepwise dissemination (sDR), stepwise transmission (sTR), and transmission efficiency (TE). CHIKV infections were performed under identical conditions to compare vector competence with TONV at 5 dpi.

The IR is defined as the number of mosquitoes with infected bodies (thorax + abdomen) out of the total number of mosquitoes. The sDR is the number of mosquitoes with infected heads relative to the number of mosquitoes with infected bodies. The sTR is defined as the number of mosquitoes with infected saliva out of those with infected heads. Transmission efficiency (TE) was defined as the proportion of mosquitoes with infectious saliva out of the total number of mosquitoes tested.

### Plaque assays

Samples were diluted 1:10 for head and body homogenates and 1/5 for saliva samples in DMEM, then inoculated onto Vero cells (90% confluence, 24-well plates). After 90 min incubation, medium was replaced with overlay (DMEM + 2% FBS + antibiotics + carboxymethylcellulose). Plates were incubated 3 days, fixed with 4% formaldehyde, and stained with 0.4% crystal violet [16].

### Molecular quantification of TONV RNA

RNA was extracted using Qiagen Viral RNA kit on BioRobot EZ1-XL. RT-qPCR was designed using CaAn 410d genome (Table 1). An IVT RNA standard was produced [16]. Amplification used GoTaq® Probe 1-Step RT-qPCR (Promega) on QuantStudioTM 3, with cycling conditions as recommended by the manufacturer and FAM-labeled probe.

**Table 1.** TONV specific primers and probe names, sequences, annealing temperatures, product sizes and target.

Name	Sequence	Tm	Product	Target
TONV_F1	GGTTCACGTTGACATCGAGG	55.9	83 bp	RTqPCR
TONV_R1	GCTTGGCTTCTACCTCAAACTG	56.0		
TONV_P1	FAM-CCTTCCTCAGAGCACTAC AACGGAGC-QSY	62.5		
TONV_NGS1F	ATGGGCGGCGTATGAGAGAAG	59.3	2934 bp	NGS PCR Fragment 1
TONV_NGS1R	GGATCTCCAGCCAAGGTCTTCC	59.5		
TONV_NGS2F	CTAGCTCAGAGCACGTCAATGTCC	59.3	2960 bp	NGS PCR Fragment 2
TONV_NGS2R	CTCTGTCCGTTCAAGTACTACCTCG	58.2		
TONV_NGS3F	GGGTTATTACTAGAGAGGAGTTCGAGGC	59.3	3048 bp	NGS PCR Fragment 3
TONV_NGS3R	CCATCACTCCGCACTGCTTCTATG	59.4		
TONV_NGS4F	GTGTCCAGGTAGGCAGAAGAGATCC	60.3	2876 bp	NGS PCR Fragment 4
TONV_NGS4R	CAGCGTGCCAATTGCTGCTG	60.3		

Note: F: Forward; R: Reverse; P: Probe; NGS: next generation sequencing; Tm: annealing temperature.

### Quasi-species sequencing

Overlapping amplicons were generated by RT–PCR (SuperScript™ IV, Thermo Fisher), purified, quantified (Qubit), and fragmented (∼250 bp). Libraries were barcoded, pooled (qPCR quantification), and sequenced on Ion S5. Reads were filtered (Q > 0.99, > 100 bp), trimmed (30 bp ends), and mapped (CLC Genomics Workbench) to CaAn 410d reference. Variants >3% frequency were analysed.

### Statistical analysis

Vector competence indices were analysed using logistic regression models in R (v4.3.3), treating each mosquito as a binary outcome (1 = infected, 0 = uninfected). For TONV, the model included days post-infection (dpi), mosquito strain (*Ae. albopictus* Réunion and IRD), and log₁₀-transformed viral dose. For TONV–CHIKV comparisons, the model included virus, log₁₀-transformed viral dose, and their interactions. Analyses of deviance were performed using the “car” package, and pairwise comparisons were assessed using estimated marginal means with Tukey’s post-hoc test (“emmeans”). Pseudo-R^2^ values (McFadden’s R^2^) were calculated from the model deviance and null deviance to quantify the proportion of deviance explained by the model. All figures were generated using “ggplot2” and “ggpubr” [17–20].

### Data availability

All data are in the manuscript. Genome sequence was deposited in GenBank (PV600894).

## Results

### Oral infection of French Ae. albopictus with TONV leads to infectious virus production in saliva

**Figure 1.**
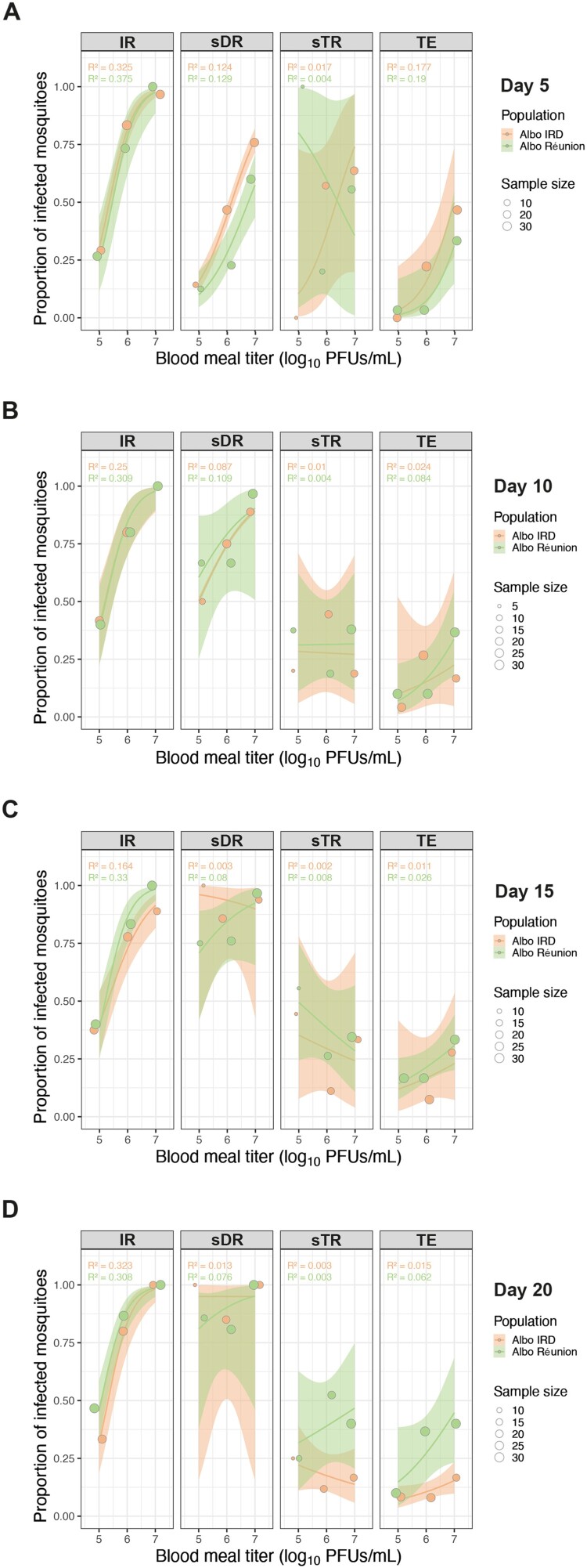
Stepwise progression of vector competence metrics for TONV in *Aedes albopictus* strains over time. *Aedes albopictus* Albo IRD (orange) and Albo Réunion (green) strains were fed infectious blood meals containing TONV at viral titers of 10^5^, 10^6^, or 10^7^ PFU/mL. Infection rate (IR), stepwise dissemination rate (sDR), stepwise transmission rate (sTR), and transmission efficiency (TE) were assessed for each strain at 5 (A), 10 (B), 15 (C), and 20 (D) days post-infection. The number of positive samples were determined by plaque assay on individual mosquito organs (body and head) and saliva. Thirty engorged mosquitoes per condition were analysed. The solid line represents the logistic regression of the data; the shaded area is the 95% confidence interval of the fit and the R^2^ values are indicated for each regression.

To assess whether *Ae. albopictus* mosquitoes from mainland France (Albo IRD) or La Réunion Island (Albo Réunion) could transmit TONV, mosquitoes were orally infected with various concentrations of TONV (10^5^ PFU/mL, 10^6^ PFU/mL, or 10^7^ PFU/mL) in the blood meal. At different time-points post-infection (5-, 10-, 15-, or 20 dpi), IR, sDR, and sTR, along with TE, were calculated. These rates were based on the presence of infectious viral particles obtained through plaque assays performed on the individual mosquito’s body, head, and secreted saliva. As early as 5 dpi, a notably short period for arbovirus transmission, the IR in mosquito bodies increased in a dose-dependent manner in both *Ae. albopictus* strains tested, with no statistically significant differences observed between them (Figure 1(A)). At a viral load of 10^5^ PFU/mL in the blood meal, the IR reached 37% for Albo IRD and 27% for Albo Réunion, increasing to 87% for Albo IRD versus 73% for Albo Réunion at 10^6^ PFU/mL (Figure 1(A)). At a blood meal viral titer of 10^7^ PFU/mL, all Albo Réunion mosquito bodies contained infectious viral particles, compared to 97% of Albo IRD bodies (Figure 1(A)). Regardless of the duration of infection (5, 10, 15, or 20 dpi), the IR increased in a dose-dependent manner, reaching nearly 100% at the highest viral dose (10^7^ PFU/mL) in both mosquito strains, with IR already peaking by day 5 and remaining consistently high through day 20 (Figure 1). Infectious dose was the only statistically significant (*p* < 0.001) predictor of IR (Table 2).

**Table 2.** Type III ANOVA results (Likelihood Ratio Tests) from logistic regression models assessing the effect of population, viral dose, time and their interactions on vector competence indices.

	Infection rate		Dissemination rate	
Effect	LR Chisq	*p-*value	LR Chisq	*p-*value
Population	0.092	0.761	1.032	0.31
LogDose	16.316	**<0.001**	9.168	**0.002**
dpi	0.11	0.74	4.961	**0.026**
Population x LogDose	0.037	0.849	1.278	0.258
Population x dpi	0.001	0.977	2.129	0.145
LogDose x dpi	0.124	0.725	2.688	0.101
Pop. x LogDose x dpi	0.038	0.846	2.221	0.136
	Transmission rate		Transmission efficiency	
Effect	LR Chisq	*p*-value	LR Chisq	*p*-value
Population	0.006	0.936	0.013	0.909
LogDose	0.619	0.432	12.334	**<0.001**
dpi	0.263	0.608	3.095	0.079
Population x LogDose	0.02	0.889	0.035	0.852
Population x dpi	0.003	0.959	0.028	0.868
LogDose x dpi	0.852	0.356	4.027	**0.045**
Pop. x LogDose x dpi	0.138	0.71	0.323	0.57

Note: dpi: days post-infection; x: interactions.

When analysing the progression of TONV within mosquito heads, it becomes clear that at 5 dpi and with a viral load of 10^5^PFU/mL, the midgut acts as a limiting barrier to the virus, as evidenced by lower sDR (9% Albo IRD and 13% Albo Réunion) compared to IR (37% Albo IRD and 27% Albo Réunion) (Figure 1(A)). For this short transmission period and low viral load, the salivary glands serve as a limiting barrier for TONV transmitted by Albo IRD (sTR of 0%) compared to Albo Réunion (sTR of 100%) (Figure 1(A)). At the same viral concentration, sTR for Albo IRD increases as the mosquito saliva collection time is delayed, reaching a peak at 15 dpi (sTR: 44%) with TE of 17%, equivalent to that obtained with Albo Réunion under the same conditions (Figure 1(B–D)).

When the blood meal is administered with a viral load of 10^5^ PFU/mL, the sDR value at 5 dpi is at 49% for Albo IRD and at 23% for Albo Réunion. At this viral concentration and time point, the salivary glands no longer act as a transmission barrier, as the sTR values (55% for Albo IRD and 20% Albo Réunion) are nearly equivalent to the sDR values (Figure 1(A)). In these conditions, the TE of Albo IRD (23%) is higher than that of Albo Réunion (3%). The difference in TE between mosquito strains (47% for Albo IRD vs 33% for Albo Réunion) is less pronounced when the viral load is 10^7^ PFU/mL (Figure 1(A)). When mosquito saliva is collected at 10 and 15 dpi, the midgut does not appear to serve as a significant barrier to viral dissemination and sDR values consistently exceed 50% irrespective of the *Ae. albopictus* strain (Figure 1(B,C)). At these two time points, the TE values between the two mosquito strains are not significantly different in all the conditions tested (Figure 1(B,C)). At the latest saliva collection time point (20 dpi), Albo Réunion shows a higher TE at viral loads of 10^6^ and 10^7^ PFU/mL (37% and 40%, respectively) compared to Albo IRD (8% and 17%, respectively) (Figure 1(D)) (*p* < 0.05)*.* Infectious dose was the only statistically significant (*p* < 0.001) predictor of IR (Table 2). Pseudo-R² values showed that IR was the most reliably predicted outcome, with R² ranging from 0.164 to 0.375, indicating a strong dose–response relationship (Figure 1). Infectious dose (*p* = 0.002) and time (*p* = 0.026) were the only statistically significant predictors of sDR (Table 2). Infectious dose (*p* < 0.001) and the interaction of infectious dose and time (*p* = 0.045) were the only statistically significant predictors of TE (Table 2). However, the explanatory power of these models varied. sDR models showed moderate to poor fit (R² = 0.003–0.129), suggesting limited influence of viral dose. sTR models consistently had low R² values (0.002–0.017), indicating that viral dose poorly predicted virus presence in saliva. TE models showed variable fits (R² = 0.011–0.190), with somewhat better predictability early in infection, but overall weak explanatory power (Figure 1).

**Figure 2.**
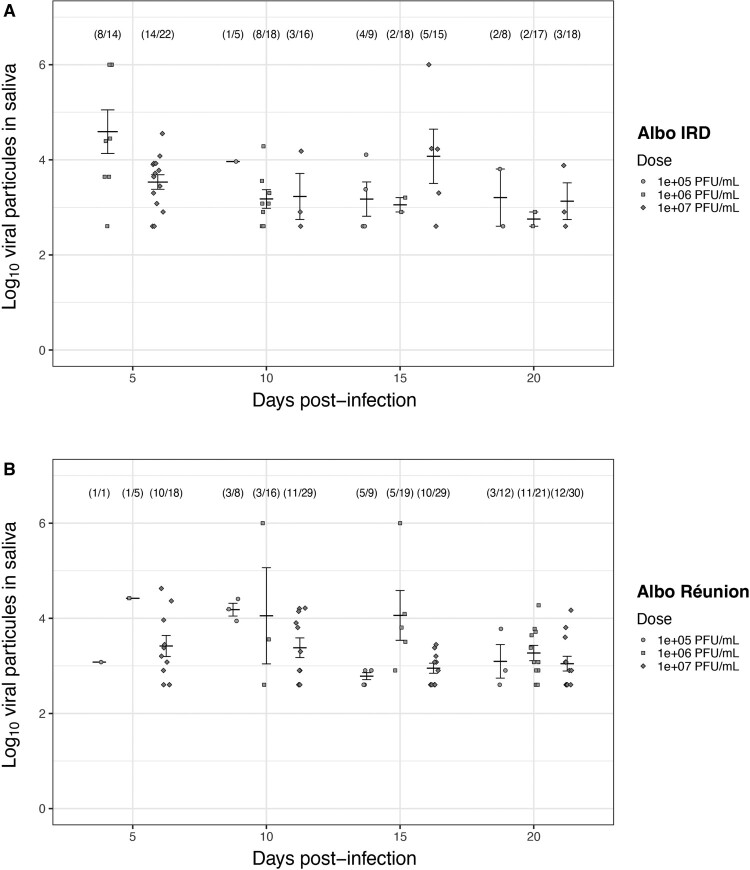
Infectious viral particles in the saliva of two orally infected *Aedes albopictus* strains with TONV. Infectious blood meals containing TONV at viral titers of 10^5^, 10^6^, or 10^7^ PFU/mL were offered to mosquitoes and viral titers in the saliva of the Albo IRD (A) and Albo Réunion (B) strains were measured at 5-, 10-, 15-, and 20- days post-infectious blood meal. Thirty engorged mosquitoes per condition were analysed. The saliva samples from individual mosquitoes with heads testing positive for TONV are shown. On Y-axis 10^6^ value was arbitrarily set as an upper limit to represent saliva samples that produced lysis plaques too numerous to count. Bars represent mean viral titers, with error bars indicating standard deviations.

The quantification of viral particles in mosquito saliva from individuals with TONV-positive heads predominantly ranges between 10² PFU/mL and 10^4^ PFU/mL as shown in Figure 2. It is also observed that some mosquitoes release high viral loads exceeding 10^6^ PFU/mL in their saliva (Figure 2). The saliva titer data reveal that both Albo IRD and Réunion are capable of excreting infectious TONV particles across a range of blood meal doses (10^5^–10^6^ PFU/mL) and time points (5-, 10-, 15-, and 20 dpi). Notably, in both *Aedes* strains, infectious viral particles were detected in the saliva as early as 5 dpi and persisted up to 20 dpi.

### Next generation sequencing of consensus and quasi-species of experimentally infected mosquito-derived samples

Various samples of TONV-infected mosquitoes were used for genome sequencing by next-generation sequencing (NGS) on an Ion Torrent platform to investigate whether viral quasi-species were associated with the differences in sDR and TE observed, at 5 dpi with a viral load of 10^6^ PFU/mL, between the mosquito strains. The panel included mosquitoes with both body and head positive by plaque assay (*n* = 3 *Ae. albopictus* Réunion and *n* = 3 *Ae. albopictus* IRD, randomly selected), mosquitoes with a positive body and a negative head (*n* = 1 *Ae. albopictus* Réunion and *n* = 1 *Ae. albopictus* IRD), and mosquitoes with both body and head negative (*n* = 1 *Ae. albopictus* Réunion and *n* = 1 *Ae. albopictus* IRD), which served as controls. The presence of TONV in the selected samples was confirmed by both RT-qPCR and plaque assays, as summarized in Table 3. Samples negative by plaque assay exhibited undetectable levels of viral RNA. In most positive samples, the quantity of viral RNA exceeded that of infectious viral particles (Table 3).

**Table 3.** Mosquito-derived organs selected for replce by NGS.

*Ae. Albopictus*	ID#	Organ	Log_10_ RNA copies/mL of homogenate	Log_10_ Plaque forming units/mL (pfu/mL)
Reunion	6	Body	10,54	7,51
		Head	6,86	6,39
Reunion	8	Body	10,36	7,09
		Head	9,44	6,05
Reunion	13	Body	10,48	7,72
		Head	8,93	5,92
Reunion	7	Body	9,56	5,64
		Head	Below limit of detection	Below limit of detection
Reunion	12	Body	Below limit of detection	Below limit of detection
		Head	Below limit of detection	Below limit of detection
IRD	5	Body	10,19	6,68
		Head	9,38	5,45
IRD	6	Body	10,39	7,18
		Head	9,32	5,51
IRD	8	Body	10,04	6,32
		Head	9,13	5,31
IRD	22	Body	8,77	4,75
		Head	Below limit of detection	Below limit of detection
IRD	17	Body	Below limit of detection	Below limit of detection
		Head	Below limit of detection	Below limit of detection

Note: Samples were obtained from Albo Réunion and Albo IRD strains that fed on blood meals containing TONV at 10^6^ PFU/mL. Dissected body and head tissues were collected at 5-days post-infection. ID# refers to the individual number assigned to each mosquito. Quantification of TONV RNA was performed by RT-qPCR and expressed as Log_10_ RNA copies per mL of homogenate. Infectious viral titers were determined by plaque assay and expressed as Log_10_ PFU/mL. Values below the limit of detection are indicated accordingly.

**Figure 3.**
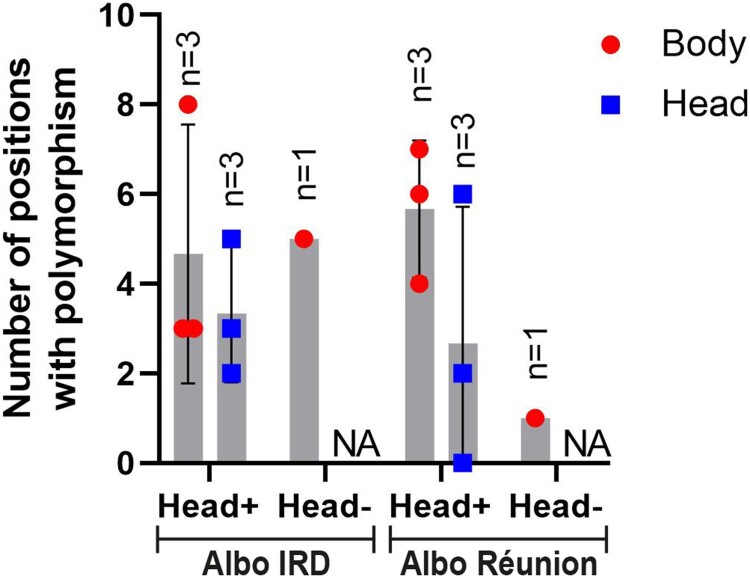
Number of polymorphic positions in the TONV genome detected in mosquito-derived samples. Whole-genome polymorphism analysis was performed on TONV-genome from body and head tissues of Albo IRD and Albo Réunion mosquitoes. Samples are grouped based on TONV RNA detection: Head + (both body and head TONV positive; *n* = 3 mosquitoes) and Head- (TONV positive body, TONV-negative head; *n* = 1 mosquito). NA = not applicable due to lack of detectable TONV RNA.

**Figure 4.**
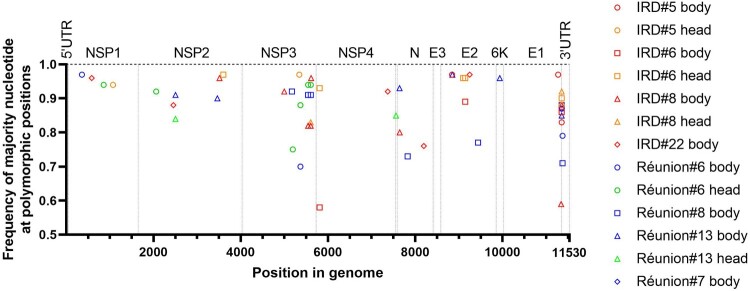
Distribution and frequency of polymorphic nucleotides across the TONV genome in mosquito-derived samples. Each point represents a polymorphic site detected in TONV-positive body or head tissues showing polymorphism from individual mosquitoes of the Albo IRD and Albo Réunion strains. The Y-axis indicates the frequency of the majority nucleotide at each polymorphic position, and the X-axis shows the genomic position of the variant. Samples include both body and head tissues, as indicated in the legend.

Samples with detectable levels of TONV RNA by RT-qPCR were subsequently used for NGS (Figure 4). The consensus genomic sequence of the TONV stock served as the reference for consensus mutation comparisons. Viral genomes from only a limited number of samples exhibited consensus mutations. TONV genomes from both the body and head of mosquito 13 (Réunion) contained one synonymous mutation (C3464T) and one non-synonymous mutation (T7640C; L to P), as compared to the reference sequence (PV600894). Viral genomes from both the body and head of mosquito 6 (Albo IRD) harboured one non-synonymous mutation (T5808A; V to E). Virus from the body of mosquito 22 (Albo IRD) displayed one synonymous mutation (T8196C), with no comparison to the head due to the absence of detectable TONV RNA. At the three polymorphic positions identified in the reference consensus sequence (PV600894), position 7656 (A/T, non-synonymous: R/S), position 8018 (A/G, non-synonymous: D/G), and position 9216 (C/T, synonymous), most mosquitoes carried identical nucleotides in both body and head. Exceptions included TONV genome of mosquito 8 (Albo IRD), which displayed different nucleotides at positions 7656 and 9216, and mosquito 6 (Albo Réunion), which showed variation at position 8018. In general, quasi-species present in each sequenced sample did not show any significant difference in the number of polymorphic positions according to mosquito strain, nor according to the tissue (Figure 3). Taken individually, polymorphism at position 11,357 showed however disparities upon mosquito strain and type of tissue. The polymorphism at this position can be observed in the viral genome sequenced from body (*n* = 3) and head (*n* = 3) of Albo IRD and body (*n* = 4) of Albo Réunion, while no polymorphism is apparent from head (*n* = 3) of Albo Réunion (Figure 4). Genomic sequences from Albo IRD with positive heads had a U/C polymorphism at this position in both body and heads parts (on average 88% of U; 12% of C), as it was observed in sequences from the bodies of Albo Réunion (on average 87% of U; 13% of C). The nucleotide at this position on viral genomes from heads Albo Réunion was a U (Figure 4).

### French Ae. albopictus mosquitoes are equally proficient at transmitting TONV and CHIKV

**Figure 5.**
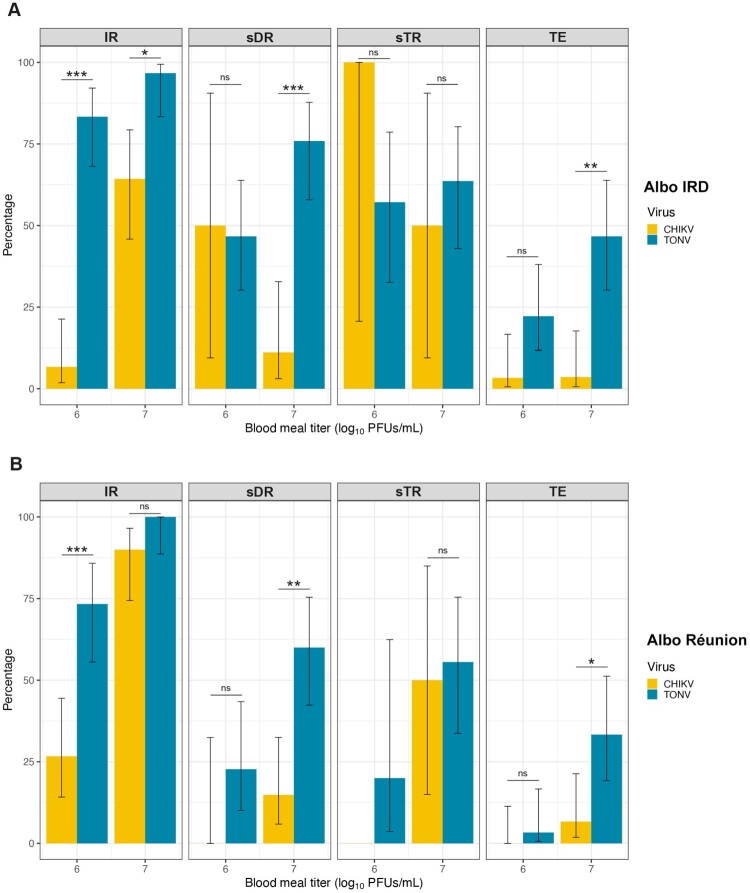
Comparison of *Aedes albopictus* vector competence for TONV and CHIKV at 5 days post-infection. Infectious blood meals containing TONV or CHIKV at viral titers of 10^6^, or 10^7^ PFU/mL were offered to mosquitoes. Thirty engorged mosquitoes per condition were analysed. Vector competence metrics of *Ae. albopictus* for CHIKV (yellow rectangles) and TONV (blue rectangles) were quantified in the Albo IRD (A) and Albo Réunion (B) organs and saliva at 5-days post-infectious blood meal. Positive samples were detected by plaque assay. Error bars are the 95% confidence intervals of the percentages. Statistical significance is denoted as follows: ns (not significant), *p < 0.05, **p < 0.01, and ***p < 0.001.

Since it is well-established that *Ae. albopictus* is capable of transmitting CHIKV shortly after a blood meal [16], we compared vector competence metrics following oral infection of mosquito strains at 5 dpi with either CHIKV (10^6^ or 10^7^ PFU/mL) or TONV (10^6^ or 10^7^ PFU/mL). The IR of TONV-infected Albo IRD was significantly higher than that observed for CHIKV-infected mosquitoes at both viral titers **(**Figure 5(A)**)**. At 10^6^ PFU/mL, TONV-infected Albo IRD achieved an IR of 87% compared to the 7% IR obtained with CHIKV-infected mosquitoes (*p* < 0.001) (Figure 5(A)). At 10^7^ PFU/mL, the IR of Albo IRD exceeded 97% TONV infection, while the IR reached approximately 64% (*p* < 0.05) in CHIKV-infected mosquitoes. The sDR also showed TONV outperforming CHIKV, particularly at 10^7^ PFU/mL, where sDR reached 76% compared to 11% in CHIKV-infected mosquitoes (*p* < 0.001). Despite the differences in the sDR of the two viruses in Albo IRD mosquitoes, no significant differences were observed in the sTR. However, the TE values obtained at a viral titer of 10^7^ PFU/mL indicate a higher TE for TONV-infected Albo IRD compared to CHIKV-infected mosquitoes (*p* < 0.01) (Figure 5(A)). The same trends were observed with the infected Albo Réunion mosquitoes (Figure 5(B)). At 10^6^ PFU/mL, TONV-infected Albo Réunion achieved an IR of 73% compared to only 27% for CHIKV-infected mosquitoes (*p* < 0.001) (Figure 5(B)). At 10^7^ PFU/mL, the IR for TONV-infected Albo Réunion reached 100%, while for CHIKV-infected mosquitoes it remained at 90% (*p* < 0.05) (Figure 5(B)). Dissemination rates in TONV-infected mosquitoes were again significantly higher at 10^7^ PFU/mL, reaching 60% compared to 7% for CHIKV-infected Albo Réunion (*p* < 0.01) (Figure 5(B)). Stepwise transmission rates values showed no significant differences between the two viruses (Figure 5(B)). However, the TE in TONV-infected Albo Réunion at 10^7^ PFU/mL was significantly higher than that obtained with CHIKV-infected Albo Réunion, which stayed below 5% (***p*** **<** **0.05**) (Figure 5(B)).

## Discussion

*Aedes albopictus* has undergone a rapid global expansion over recent decades, primarily driven by international trade and its remarkable ecological adaptability13 [13,21,22]. Now established in parts of Europe, the Americas, Africa, and numerous islands in the Indian Ocean, it has become a major concern for public health authorities due to its competence for transmitting a broad range of arboviruses, such as dengue virus (DENV), CHIKV, and Zika virus (ZIKV) [23–25]. Its documented involvement in several arboviral outbreaks, including the explosive CHIKV epidemic on La Réunion in 2005–2006, has highlighted its capacity to support urban transmission cycles [26]. In this context, we investigated the potential of *Ae. albopictus* populations to transmit TONV, an emerging South American alphavirus. Although human cases of TONV infection have thus far been limited to Latin America, recent clinical evidence has described neurological and congenital manifestations, including a confirmed case of vertical transmission associated with severe fetal abnormalities such as cerebral necrosis and spinal cord lesions [6,27].

Our findings demonstrate that *Ae. albopictus* populations from mainland France and La Réunion Island can efficiently transmit TONV, with infectious virus detected in saliva as early as five days post-infectious blood meal, indicating a short extrinsic incubation period and high epidemic potential. In addition, TONV titers in the mosquito saliva were in the range of those typically observed in vector competence assays involving arboviruses [16,25]. This is particularly concerning, as the transmission dynamics closely mirror those of CHIKV, which has caused widespread epidemics in similar settings [24].

Notably, TE for TONV was comparable or higher than CHIKV under identical conditions, implying intrinsic viral traits enabling effective replication and dissemination through *Ae. albopictus* barriers. While both *Ae. albopictus* strains supported efficient TONV transmission, some temporal differences were observed. In the mosquito from mainland France, TE decreased over time, whereas it progressively increased in the La Réunion strain. These differences may reflect intrinsic variation in immune responses, microbiota composition, or midgut escape efficiency [28].

In our study, the TONV quasi-species present in each sequenced mosquito sample did not show any significant difference in the number of polymorphic positions according to mosquito strain, body part, or the TONV RNA status of the head. Positions displaying polymorphism are frequent in the 3′ half of the *nsP3* coding region, suggesting this region exhibits greater genomic plasticity compared to other parts of the genome. Similar patterns have been observed in CHIKV and VEEV, especially within the C-terminal hypervariable region of *nsP3*, where they have been linked to enhanced viral fitness, modulation of host immune responses, or improved adaptation to vectors [26,29,30]. The presence of numerous polymorphic sites within the 3′ region of the *nsP3* gene of the TONV genome, observed in both bodies and heads, suggests a lack of strong selective pressure on these variable positions. This observation appears to apply to the entire genome, with the exception of position 11,357, located in the 3′ untranslated region (3′UTR). Polymorphism at position 11,357 was detected in most body samples but absent from heads of Albo Réunion, but not from heads of Albo IRD. The 3′UTR is critical for RNA stability and replication efficiency and is known to evolve under host-specific constraints [31]. The absence of variability in head tissues of Albo Réunion may reflect a bottleneck or selective barrier during viral dissemination to salivary glands, a selection mechanism apparently absent in Albo IRD. This observation warrants further investigation as sequencing data were obtained for only n = 3 mosquitoes per strain, and expanding the sequencing to include larger sample size would provide a more comprehensive view of intra-vector viral diversity, as similarly observed in other alphaviruses. Chikungunya virus is generally characterized by a highly conserved genome shaped by strong purifying selection, ensuring genomic stability across hosts and vectors, it has occasionally acquired adaptive mutations, such as the well-known E1-A226 V substitution, which enhanced replication and transmission in *Ae. albopictus* during the 2005–2006 La Réunion outbreak [26]. Despite strong purifying selection on alphavirus genomes, specific ecological conditions or vector interactions may support adaptive variants. TONV demonstrates high transmissibility and a possible genetic plasticity in *Ae. albopictus*, suggesting a risk of emergence in Europe, where this vector is widespread. The establishment of *Ae. albopictus* in both tropical and temperate regions underscores the critical importance of assessing its vector competence for emerging arboviruses. In August 2024, CHIKV re-emerged on Réunion Island, 18 years after the initial major outbreak, with genomic analyses of 173 viral sequences revealing a monophyletic lineage harbouring the E1 A226 V mutation, known to enhance adaptation to *Ae. albopictus* [16,26]. The ongoing CHIKV outbreak on La Réunion (>33,000 cases, multiple deaths in 2025) [24,32] highlights the need for vigilance. Genomic surveillance and vector competence studies are critical to anticipate TONV's zoonotic emergence, as adaptive mutations, particularly in the 3′ untranslated region (3′UTR), may enhance viral fitness in mosquitoes and signal increased epidemic potential. These findings, alongside confirmed travel-related and autochthonous CHIKV cases in mainland France and Italy [33–40], clearly demonstrate that, *Ae. albopictus* can sustain its local transmission in temperate climates. Given the circulation of TONV in French Guiana and increasing travel connections, the introduction of TONV into mainland France is a plausible scenario. As observed with CHIKV, *Ae. albopictus* could potentially serve as an efficient vector for local transmission [16].

This study, while informative, has some inherent constraints and limits. All experiments were performed at a constant temperature of 28°C, which reflects tropical conditions and provides useful baseline data. However, this does not fully account for the lower and more variable temperatures typically encountered in temperate regions such as mainland France, where *Ae. albopictus* is also established. Incorporating a broader range of temperatures (e.g. 20–25 °C) in future studies would help refine our understanding of the potential for TONV transmission under more diverse environmental conditions, including variations in vector spectrum using field-collected European early-generation *Ae. albopictus*, *Culex pipiens*, *Anopheles atroparvus* mosquitoes or the possibility of vertical transmission. Considering the ecological adaptability of *Ae. albopictus* in temperate regions, partly linked to its efficient lipid metabolism and capacity for egg dormancy [14], it may be worth exploring whether vertical transmission of TONV could occur in this species. Such a mechanism might facilitate viral persistence during unfavourable seasons and contribute to overwintering in regions with colder climates.

Additionally, only a single strain of TONV and CHIKV was used in this study, which may limit the generalizability of our findings, given the known influence of specific virus-vector genotype interactions [41]. The TONV strain used also originates from an earlier period; evaluating currently circulating strains would offer a more accurate perspective on viral fitness and potential transmission dynamics. Integrative studies conducted in French Guiana, in line with One Health principles, are also essential to elucidate the role of vertebrate hosts in the TONV transmission cycle, particularly those that may serve as animal reservoirs. In this context, investigating whether *Ae. albopictus* could act as a bridge vector is particularly relevant, as its ecological flexibility and feeding behaviour may facilitate viral transmission between animals and humans across diverse habitats [14,22].
